# A molecular and immunohistochemical study of 37 cases of ovarian Sertoli–Leydig cell tumor

**DOI:** 10.1007/s00428-024-03984-5

**Published:** 2024-11-27

**Authors:** Kristýna Němejcová, Nikola Hájková, Eva Krkavcová, Michaela Kendall Bártů, Romana Michálková, Adam Šafanda, Marián Švajdler, Tetiana Shatokhina, Jan Laco, Radoslav Matěj, Jitka Hausnerová, Jozef Škarda, Monika Náležinská, Tomáš Zima, Pavel Dundr

**Affiliations:** 1 Department of Pathology, First Faculty of Medicine, Charles University and General University Hospital in Prague, Prague, Czech Republic; 2 Šikl’s Department of Pathology, The Faculty of Medicine and Faculty Hospital in Pilsen, Charles University, Pilsen, Czech Republic; 3 Department of Oncological Pathology, Masaryk Memorial Cancer Institute, Brno, Czech Republic; 4 The Fingerland Department of Pathology, Charles University Faculty of Medicine in Hradec Králové and University Hospital, Hradec Králové, Czech Republic; 5 Department of Pathology, 3rd Faculty of Medicine, Charles University, University Hospital Královské Vinohrady, 10034 Prague, Czech Republic; 6 Department of Pathology and Molecular Medicine, Third Faculty of Medicine, Charles University, Thomayer University Hospital, Prague, Czech Republic; 7 Department of Pathology, University Hospital Brno and Medical Faculty, Masaryk University, Brno, Czech Republic; 8 Department of Pathology, University Hospital Ostrava and Faculty of Medicine University of Ostrava, Ostrava, Czech Republic; 9 Division of Gynecologic Oncology, Department of Surgical Oncology, Masaryk Memorial Cancer Institute and Medical Faculty of Masaryk University, Brno, Czech Republic; 10 Institute of Medical Biochemistry and Laboratory Diagnostics, First Faculty of Medicine, Charles University and General University Hospital in Prague, Prague, Czech Republic

**Keywords:** Ovarian tumors, Sertoli–Leydig cell tumor, Sex cord-stromal tumor, Immunohistochemistry, DICER1, MRNA expression

## Abstract

This study provides an analysis of 37 ovarian Sertoli–Leydig cell tumors (SLCT), focusing on their morphological, immunohistochemical, and molecular features. The cohort was comprised of 9 well-differentiated, 25 moderately differentiated, and 3 poorly differentiated tumors. The immunohistochemical analysis was performed with 28 markers, including diagnostic markers and markers with possible predictive significance. The results showed high expression of sex cord markers (FOXL2, SF1, inhibin A, CD99, calretinin, ER, PR, AR), and variable expression of other markers such as CKAE1/3 (83%), CAIX (14%), and MUC4 (1%). Loss of PTEN expression was present in 14% of cases, and CTLA4 expression was seen in 43% of cases. All tumors were MMR proficient and HER2 and PD-L1 negative. The molecular analysis showed *DICER1* mutations in 54.5% of cases, and a *FOXL2* mutation in 6% of tumors. In addition, we detected 2 cases with *TERT* promoter mutation. RNA NGS sequencing identified significant differences in mRNA expression between *DICER1*^MUT^ and *DICER1*^WT^ tumors. The *DICER1*^WT^ tumors showed increased expression of *PRKCA*, *HNF1A*, *LDLR*, and *MAP2K5.* On the contrary, the *DICER1*^MUT^ cases showed increased expression of *CDK6*, *NOTCH2*, and *FGFR2*. The results of our study show that SLCTs exhibit distinct molecular features based on their degree of differentiation. We have confirmed that *DICER1* mutations are characteristic of moderately and poorly differentiated SLCTs, while well-differentiated SLCTs may represent a distinct entity. *DICER1*^MUT^ and *DICER1*^WT^ tumors showed different mRNA expression profiles. The *FOXL2* mutation is less common in these tumors and is mutually exclusive with the *DICER1* mutation.

**Supplementary Information:**

The online version contains supplementary material available at 10.1007/s00428-024-03984-5.

## Introduction

Sertoli–Leydig cell tumors (SLCT) are rare ovarian sex cord-stromal tumors that account for less than 0.5% of all ovarian neoplasms [1]. They typically affect young patients (mean age of 25 years) and may present with androgenic symptoms and/or as an ovarian mass. Histologically, according to the current WHO Classification, SLCTs are divided into three main subtypes which reflect their prognosis: well-differentiated, moderately differentiated, and poorly differentiated [1, 2]. Retiform SLCT can be regarded as a fourth type.

Regarding the molecular features, somatic or germline mutations of the *DICER1* gene have been found in some tumors [3]. SLCTs mostly occur sporadically, but can also develop as a part of the DICER1 syndrome in cases of germline mutations. Besides SCLT, this syndrome is characterized by cervical rhabdomyosarcoma and unusual tumors of the lung, thyroid gland, and/or kidney [1, 4, 5]. The other, less common, mutation that can be found in SLCT is the *FOXL2* mutation c.402C > G, p.(Cys134Trp), which is mutually exclusive with *DICER1*. The *FOXL2* mutation accounts for 0–22% of SLCT and may cause estrogenic manifestations [1, 2, 6, 7].

Based on the presence and/or absence of the relevant mutations, SLCT can be divided into three molecular subtypes, which also reflect their clinicopathological features: *DICER1* mutant, *FOXL2* mutant, and *DICER1/FOXL2* wild type [2, 8]. So far, the *DICER1* mutation has been found only in moderately/poorly differentiated tumors. Patients with *DICER1* mutations tend to be younger and often show androgenic manifestations. The *FOXL2* mutation has also been described only in moderately/poorly differentiated tumors, but patients in this group are typically older (postmenopausal), and the tumors present with estrogenic effects. The *DICER1/FOXL2* wild-type group represents patients of intermediate age and comprises well-differentiated tumors only [2]. Several recent studies have suggested that based on the morphological features and molecular results, well-differentiated SLCTs probably represent a different tumor entity from moderately and poorly differentiated SLCTs. These two often comprise a morphologic spectrum with an admixture of both variants, including the presence of a retiform component, compared to the well-differentiated type which typically occurs in pure form [2, 9].

In our study, we focused on the immunohistochemical and molecular characterization of 37 SLCT. The antibodies used included 12 of the “diagnostic” antibodies already examined in previously published studies, although some of those were only on a limited number of cases [7, 10–19]. We also analyzed 9 other “diagnostic” antibodies and 7 antibodies with possible predictive significance, the expression of which has not yet been described in SCLT, including the expression of HER2, PD-L1, CTLA4, and mismatch repair (MMR) proteins. The molecular analysis in all tumors included DNA and RNA NGS sequencing. To the best of our knowledge, our study represents the first study focusing also on the mRNA expression pattern of SLCT.

## Methods

### Samples

The cases were selected from the archives of the cooperating institutions as a part of a project focusing on sex cord-stromal tumors. In total, 39 tumors diagnosed as SLCT, and 5 cases of SLCT originally diagnosed as adult granulosa cell tumors (AGCT) were submitted to this project, including 23 consultation cases, and 21 cases that came from the routine practice of the cooperating institutions. All cases were reviewed by two pathologists with expertise in gynecopathology (KN and PD). During the central review, 5 cases originally diagnosed as SLCT were reclassified as other tumors (1 AGCT, 1 juvenile granulosa cell tumor, 1 sex cord tumor with annular tubules (with *STK11* gene mutation), 2 sex cord-stromal tumors NOS), and excluded from the study. Two other cases were excluded from the study, one due to the suboptimal tissue quality, and in one case there was not enough material for subsequent analysis. The determination of tumor differentiation was based on the degree of tubular differentiation of the Sertoli cell component, maturity of Sertoli cells, degree of cytologic atypia and number of mitotic figures, and arrangement and quantity of Leydig cells into well-differentiated (G1), moderately differentiated (G2), and poorly differentiated (G3) [20].

The final sample set consists of 37 cases, including 9 well-differentiated, 25 moderately differentiated, and 3 poorly differentiated tumors.

The morphological aspects of all the tumors including the mitotic rate, presence of sarcomatoid and/or heterologous elements, and presence of lymphovascular invasion (LVSI) were assessed using whole-tissue sections.

Molecularly, 33 cases were successfully tested by NGS DNA and 22 cases by NGS RNA. All 37 tumors were eligible for mRNA expression profiling. The clinicopathological characteristics of the 37 cases are summarized in Table 1.

**Table 1:** Characterization of the dataset of 37 patients with SLCT

Characteristics	SLCTs *n* (%)
Age at diagnosis (years)	
Mean (SD)	42 (20.4)
Median (range)	43 (14–76)
Grade	
Well-differentiated (G1)	9 (24%)
Moderately differentiated (G2)	25 (68%)
Poorly differentiated (G3)	3 (8%)
FIGO (n/a = 26)	
IA	8 (67%)
IC1	3 (25%)
IIB	1 (8%)
Recurrences (n/a = 31)	
No	6 (86%)
Yes	1 (14%)
Mitoses (mitoses/HPF)	
Mean (SD)	3.4 (3.9)
Median (range)	2 (0–15)
Retiform component	
No	36 (95%)
Yes	2 (5%)
Sarcomatoid component	
No	36 (95%)
Yes	2 (5%)
Heterologous component	
No	35 (92%)
Yes	3 (8%)

Percentages are counted only from the available data and are rounded up/down; mitoses/HPF, mitoses per 10 high-power fields

*SLCT* Sertoli–Leydig cell tumor, *SD* standard deviation, *n/a* data not available

### Immunohistochemical analysis

The immunohistochemical (IHC) analysis was performed using 4-µm thick sections of formalin-fixed and paraffin-embedded (FFPE) tissue using tissue microarrays (TMAs). The eligible areas of each tumor were selected, and two tissue cores (each 2 mm in diameter) were taken from the donor block using the tissue microarray instrument TMA Master (3DHISTECH Ltd., Budapest, Hungary). The antibodies used included the “diagnostic” markers (FOXL2, SF1, CD99, inhibin A, calretinin, ER, PR, AR, p53, p16, Ki67, and CKAE1/3), new markers which have not yet been analyzed in SLCT (PTEN, CAIX (carbonic anhydrase IX), DPC4, GATA3, napsin A, ARID1A, SATB2, MUC4, and TTF1), and selected predictive markers (CTLA4, PD-L1, HER2, MLH1, PMS2, MSH2, and MSH6). The list of their manufacturers, clones, and dilutions is provided in Supplementary Table S1.

The expression of all markers was double-blindly evaluated by two pathologists (KN, AŠ). Cases were classified based on the overall percentage of positive tumor cells as negative (entirely negative or < 5% of positive tumor cells) or positive (≥ 5% of positive tumor cells), except for p53, p16, Ki67, HER2, and PD-L1. The p53 protein expression was assessed as either the “wild-type” or “aberrant type”. The “aberrant type” of staining was defined as diffuse intense nuclear positivity of > 80% of tumor cells, cytoplasmic p53 positivity, or the complete absence of staining with positive internal control (the so-called null pattern) [21]. The expression of p16 was regarded as block-positive (diffuse staining of tumor cells in the nuclei and/or cytoplasm), or negative (focal/patchy or absent staining). Ki67 was assessed as a continuous variable based on the proportion of positive tumor cells (0–100%). It was counted manually in 200 tumor cells in the hot-spots, or in randomly selected fields in cases of homogenous expression. For ARID1A, MMR, PTEN, and DPC4 the loss of expression in tumor cells with retained staining in stromal cells was evaluated (loss of expression was defined as less than 5% of positive tumor cells). HER2 scoring was performed in accordance with the 2018 ASCO Guidelines for breast carcinoma, as there is currently no established scoring system for ovarian tumors [22]. PD-L1 expression was evaluated as the percentage of positive tumor cells (tumor proportion score; TPS). Only occasional rare lymphocytes were present in the stroma of a few cases, so neither CTLA4 expression in immune cells nor PD-L1 combined positive score (CPS) could be assessed.

### Molecular analysis

Genomic DNA and total RNA were isolated from the FFPE tissue from the tumor using the Quick-DNA/RNA FFPE Miniprep Kit (Zymo Research) according to the manufacturer’s protocol. DNA was extracted also from the adjacent non-neoplastic tissue (Magcore Genomic DNA FFPE One step kit; RBC Bioscience) for sequencing analysis to rule out a potential germline origin of the *DICER1* mutations detected in the tumor.

Sequence capture NGS analysis of DNA was performed using the KAPA HyperPlus kit according to KAPA HyperCap Workflow v3.0 (Roche) and a panel of hybridization probes against multiple targets of cancer-relevant genes (Supplementary Table 2; 788 genes or gene parts; 2440 kbp of target sequence including 1992 kbp of coding regions; Roche). The prepared sample libraries were pair-end sequenced by the NextSeq 500 instrument (Illumina) using NextSeq 500/550 High Output Kit v2.5 (Illumina). The biostatistical evaluation was performed using the CLC Genomics Workbench software (CLC GW; Qiagen, Venlo, The Netherlands). The interpretation of DNA variants, calculation of tumor mutation burden (TMB), and status of microsatellite instability were determined as previously described [23, 24]. The quality and state of the DNA isolated from the FFPE tissues varied across the samples and was of insufficient quality for CNV assessment.

The total RNA samples were processed according to the KAPA RNA HyperPrep Kit protocol, described in more detail in our previous study [23]. The target sequences were enriched by the standard KAPA HyperCap Workflow v3 (Roche) using a custom panel focused on the pan-cancer markers and potential fusion genes (Supplementary Table 2; 247 genes; 675 kbp of the target DNA sequence; Roche).

All SLCT cases were eligible for expression profiling, which was conducted using targeted RNA-Seq expression analysis (RNA-Seq Analysis module). The detection of gene fusions was performed by the CLC GW Detect and Refine Fusion Genes module. Only genes with a transcript per million (TPM) value above 60 were evaluated in the gene expression analyses. The “Differential Expression in Two Groups” module in GW was used to analyze RNA differences between the group of tumors with detected mutation in *DICER1* (*DICER1*^MUT^) and *DICER1* wild-type (*DICER1*^WT^). A Bonferroni correction test at the level of 0.05 or less was considered a significant difference. The TMP values of mRNA were normalized to the housekeeping gene *HPRT1*.

### Statistical analyses

Standard descriptive statistics were employed to summarize the data. Categorical variables were described using the absolute and relative frequencies, continuous variables were described as the mean with standard deviation (SD) or median with interquartile range. Differences in the expression of IHC markers between grades of differentiation (well-differentiated vs. moderately and poorly differentiated) were analyzed using Fisher’s Exact test or the Mann–Whitney *U* test as appropriate. The association between the mutation status of *DICER1* and/or *FOXL2* and tumor grade was assessed using Fisher’s exact test.

## Results

All cases showed the typical morphological features of SLCT. Briefly, all well-differentiated tumors consisted of well-formed hollow and solid tubules of columnar cells with round nuclei, with Leydig cell clusters between tubules. No necroses were present. Moderately differentiated cases showed mostly lobular patterns on low power; and consisted of immature Sertoli cells with predominantly solid patterns with areas of trabecular or nested formations (9/25), cords/trabecular formations (8/25), nested/alveolar arrangement (7/25), and cystic/tubular morphology (1/25). Leydig cells were mostly present in clusters at the periphery of the lobules. Two cases showed heterologous elements (mucinous epithelium), and one had a retiform component. Necroses were noticed in two cases, and nucleoli were clearly visible in 20/25 cases, in eleven of those only focally. All poorly differentiated cases showed a mostly solid arrangement of immature Sertoli cells, in 2/3 cases with a sarcomatoid component, and in 1/3 with a retiform component. The heterologous elements (rhabdomyoblastic) and necrosis were present in one case.

Both SLCT with *FOXL2* mutation (see below) were moderately differentiated cases with typical features of SLCT, one with a predominantly solid pattern with areas of trabecular formations, and the second showed a mostly nested arrangement.

The mitotic rate ranged from 0 to 15 mitoses/10 high-power fields (HPF). The median of the full cohort was 2 mitoses/ 10 HPF (mean = 3.4 ± 3.9). There was a slight difference between the well-differentiated and moderately/poorly differentiated subgroups, with a lower mitotic rate observed in the well-differentiated cases (median = 1, mean = 2.1 ± 3.4) compared to the moderately/poorly differentiated cases (median = 2, mean = 3.9 ± 4.1; Mann–Whitney *U* test, *U* = 79, *Z* = 1.99, *p* = 0.04). No case showed LVSI.

**Fig. 1:**
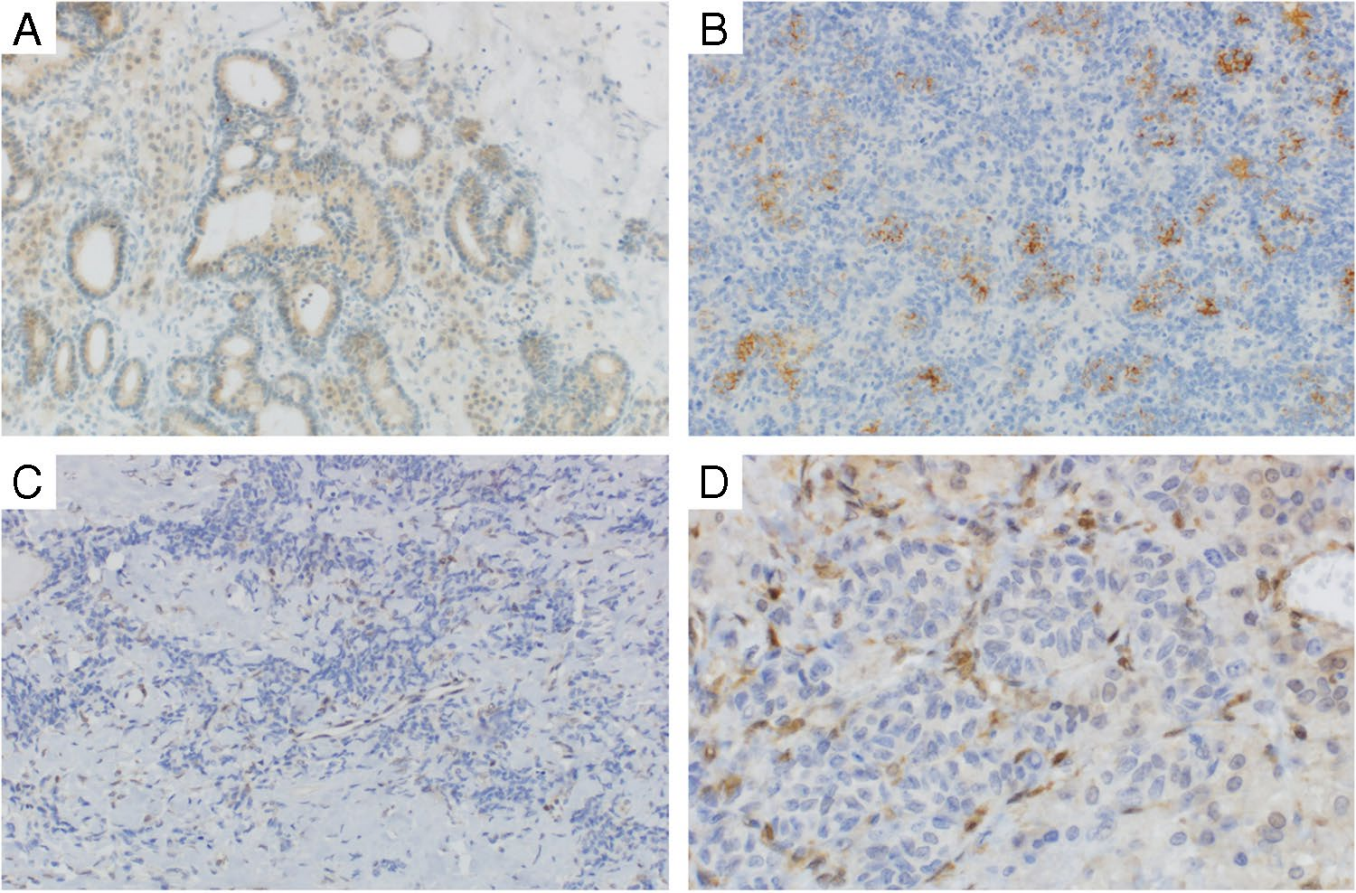
**A** CTLA4 expression (200 ×), **B** MUC4 expression (200 ×), **C** loss of PTEN expression (200 ×), and **D** loss of PTEN expression (400 ×)

The results of the immunohistochemical analyses are summarized in Tables 2 and 3 (see also Fig. 1). Briefly, FOXL2, SF1, inhibin A, CD99, calretinin, ER, PR, AR, CKAE1/3 showed expression in 97%, 97%, 94%, 81%, 58%, 72%, 58%, 67%, and 83% of cases. CAIX was positive in 14% of cases (5/37) and was the only marker (the expression of which was categorized as positive or negative) to have differed significantly with tumor differentiation. Specifically, 56% (5/9) of the well-differentiated cases were CAIX positive; two in less than 10% of tumor cells, and three with expression in more than 85% of tumor cells. While no positive cases were detected in the moderately/poorly differentiated group (*p* < 0.001). GATA3, SATB2, napsin A, and TTF1 were completely negative in all cases. One case was MUC4 positive in 55% of tumor cells, showing mostly weak to moderate intensity of staining. This case was moderately differentiated and showed the expression of FOXL2, SF1, inhibin A, CD99, calretinin, and hormonal receptors. EMA staining was negative.

**Table 2:** Overview of the percentage of positivity and ratio of positive and negative events in selected IHC markers in SLCT

Marker		Marker		Marker	
**FOXL2**		p53*		**CTLA4**	
Median (range)	90 (0–100)	Median (range)	n/a	Median (range)	0 (0–90)
Mean (SD)	77 (29.6)	Mean (SD)	n/a	Mean (SD)	22 (31.2)
No. of positive cases	35 (97%)	No. of positive cases	0 (0%)	No. of positive cases	16 (43%)
No. of negative cases	1 (3%)	No. of negative cases	35 (100%)	No. of negative cases	21 (57%)
SF–1		p16*		**PTEN**	
Median (range)	100 (0–100)	Median (range)	n/a	Median (range)	75 (0–100)
Mean (SD)	97 (16.6)	Mean (SD)	n/a	Mean (SD)	63 (36.4)
No. of positive cases	35 (97%)	No. of positive cases	0 (0%)	No. of positive cases	30 (86%)
No. of negative cases	1 (3%)	No. of negative cases	37 (100%)	No. of negative cases	5 (14%)
**CD99**		**GATA3**		**HER2**	
Median (range)	73 (0–100)	Median (range)	0 (–)	Median (range)	0 (–)
Mean (SD)	58 (36.9)	Mean (SD)	0 (–)	Mean (SD)	0 (–)
No. of positive cases	29 (81%)	No. of positive cases	0 (0%)	No. of positive cases	0 (0%)
No. of negative cases	7 (19%)	No. of negative cases	37 (100%)	No. of negative cases	37 (100%)
**Inhibin**		**ARID1A**		**PD–L1**	
Median (range)	62 (0–100)	Median (range)	100 (70–100)	Median (range)	0 (–)
Mean (SD)	56 (38.2)	Mean (SD)	99 (5.2)	Mean (SD)	0 (–)
No. of positive cases	34 (94%)	No. of positive cases	36 (100%)	No. of positive cases	0 (0%)
No. of negative cases	2 (6%)	No. of negative cases	0 (0%)	No. of negative cases	37 (100%)
**Calretinin**		**Napsin A**		**MLH1**	
Median (range)	9 (0–98)	Median (range)	0 (–)	Median (range)	100 (65–100)
Mean (SD)	24 (29.5)	Mean (SD)	0 (–)	Mean (SD)	96 (7.8)
No. of positive cases	21 (58%)	No. of positive cases	0 (0%)	No. of positive cases	36 (100%)
No. of negative cases	15 (42%)	No. of negative cases	36 (100%)	No. of negative cases	0 (0%)
**ER**		**SATB2**		**PMS2**	
Median (range)	53 (0–100)	Median (range)	0 (–)	Median (range)	100 (65–100)
Mean (SD)	44 (37.3)	Mean (SD)	0 (–)	Mean (SD)	98 (6.0)
No. of positive cases	26 (72%)	No. of positive cases	0 (0%)	No. of positive cases	36 (100%)
No. of negative cases	10 (28%)	No. of negative cases	35 (100%)	No. of negative cases	0 (0%)
**PR**		**MUC4**		**MSH2**	
Median (range)	12 (0–98)	Median (range)	0 (0–35)	Median (range)	100 (80–100)
Mean (SD)	28 (34.7)	Mean (SD)	1 (5.8)	Mean (SD)	99 (3.8)
No. of positive cases	21 (58%)	No. of positive cases	1 (3%)	No. of positive cases	36 (100%)
No. of negative cases	15 (42%)	No. of negative cases	35 (97%)	No. of negative cases	0 (0%)
**AR**		**TTF1**		**MSH6**	
Median (range)	31 (0–99)	Median (range)	0 (–)	Median (range)	100 (85–100)
Mean (SD)	41 (39.4)	Mean (SD)	0 (–)	Mean (SD)	99 (2.6)
No. of positive cases	24 (67%)	No. of positive cases	0 (0%)	No. of positive cases	36 (100%)
No. of negative cases	12 (33%)	No. of negative cases	36 (100%)	No. of negative cases	0 (0%)
**CKAE1/3**		**DPC4**			
Median (range)	68 (0–100)	Median (range)	12 (0–100)		
Mean (SD)	61 (38.9)	Mean (SD)	29 (34.4)		
No. of positive cases	30 (83%)	No. of positive cases	26 (79%)		
No. of negative cases	6 (17%)	No. of negative cases	7 (21%)		
**Ki67**		**CAIX**			
Median (range)	3 (0–38)	Median (range)	0 (0–97)		
Mean (SD)	8 (9.4)	Mean (SD)	8 (24.7)		
No. of positive cases	n/a	No. of positive cases	5 (14%)		
No. of negative cases	n/a	No. of negative cases	32 (86%)		

The cut-off for positive/negative case is 5% (methods section)

*IHC* immunohistochemical, *SD* standard deviation, *n/a* not available

^*^In the case of p53, aberrant cases are marked as positive, wild-type cases are marked as negative

^*^In the case of p16, negative and focal cases are marked as negative, diffusely positive cases are marked as positive

**Table 3:** Association between the expression of IHC markers and differentiation of tumors (well-differentiated SLCT vs moderately/poorly differentiated tumors)

Marker	Grade 1	Grades 2–3	*p*-value	Marker	Grade 1	Grades 2–3	*p*-value	Marker	Grade 1	Grades 2–3	*p*-value
**FOXL2**			> 0.995	p53*			NULL	**CTLA4**			0.135
Positive	9 (100%)	26 (96%)		Positive	0 (0%)	0 (0%)		Positive	6 (67%)	10 (36%)	
Negative	0 (0%)	1 (4%)		Negative	9 (100%)	26 (100%)		Negative	3 (33%)	18 (64%)	
SF1			> 0.995	p16*			NULL	**PTEN**			> 0.995
Positive	9 (100%)	26 (96%)		Positive	0 (0%)	0 (0%)		Positive	8 (89%)	22 (85%)	
Negative	0 (0%)	1 (4%)		Negative	9 (100%)	28 (100%)		Negative	1 (11%)	4 (15%)	
**CD99**			0.652	**GATA3**			NULL	**HER2**			NULL
Positive	8 (89%)	21 (78%)		Positive	0 (0%)	0 (0%)		Positive	0 (0%)	0 (0%)	
Negative	1 (11%)	6 (22%)		Negative	9 (100%)	28 (100%)		Negative	9 (100%)	28 (100%)	
**Inhibin**			> 0.995	**ARID1A**			NULL	**PD-L1**			NULL
Positive	9 (100%)	25 (93%)		Positive	9 (100%)	27 (100%)		Positive	0 (0%)	0 (0%)	
Negative	0 (0%)	2 (7%)		Negative	0 (0%)	0 (0%)		Negative	9 (100%)	26 (100%)	
**Calretinin**			0.443	**Napsin A**			NULL	**MLH1**			NULL
Positive	4 (44%)	17 (63%)		Positive	0 (0%)	0 (0%)		Positive	9 (100%)	27 (100%)	
Negative	5 (56%)	10 (37%)		Negative	9 (100%)	27 (100%)		Negative	0 (0%)	0 (0%)	
**ER**			0.392	**SATB2**			NULL	**PMS2**			NULL
Positive	8 (89%)	18 (67%)		Positive	0 (0%)	0 (0%)		Positive	9 (100%)	27 (100%)	
Negative	1 (11%)	9 (33%)		Negative	9 (100%)	27 (100%)		Negative	0 (0%)	0 (0%)	
**PR**			0.438	**MUC4**			> 0.995	**MSH2**			NULL
Positive	4 (44%)	18 (64%)		Positive	0 (0%)	1 (4%)		Positive	9 (100%)	27 (100%)	
Negative	5 (56%)	10 (36%)		Negative	9 (100%)	26 (96%)		Negative	0 (0%)	0 (0%)	
**AR**			0.432	**TTF1**			NULL	**MSH6**			NULL
Positive	5 (56%)	20 (71%)		Positive	0 (0%)	0 (0%)		Positive	9 (100%)	27 (100%)	
Negative	4 (44%)	8 (29%)		Negative	9 (100%)	27 (100%)		Negative	0 (0%)	0 (0%)	
**CKAE1/3**			0.302	**DPC4**			> 0.995				
Positive	9 (100%)	22 (79%)		Positive	6 (75%)	20 (80%)					
Negative	0 (0%)	6 (21%)		Negative	2 (25%)	5 (20%)					
**Ki67**			0.221	**CAIX**			** < 0.001**				
Mean (SD)	3.5 (5.3)	9.5 (10.1)		Positive	5 (56%)	0 (0%)					
Median	2	7		Negative	4 (44%)	28 (100%)					

NULL, analysis not possible due to the absence of either positive or negative cases in the comparison groups; *p*-values are based on Fisher´s exact tests, except for Ki67, which is based on Mann–Whitney *U* test. Significant *p*-values are indicated in bold. The cut-off for positive/negative case is 5% (“Methods” section)

*IHC* immunohistochemical, *SD* standard deviation

^*^In the case of p53, aberrant cases are marked as positive, wild-type cases are marked as negative

^*^In the case of p16, negative and focal cases are marked as negative, diffusely positive cases are marked as positive

PTEN showed loss of expression in 14% (5/35) of cases, 3 moderately differentiated, and one poorly and one well-differentiated case. DPC4 expression was lost in 21% (7/33) of cases. No case showed the aberrant staining pattern of p53 or diffuse (block) positivity of p16. The expression of Ki67 showed a median value of 3 (range 0–38, mean = 8 ± 9.4). There was a higher Ki67 rate in the moderately/poorly differentiated cases with a median of 7% (mean = 9.5 ± 10.1%) compared to the well-differentiated subgroup with a median of 2% (mean = 3.5 ± 5.3%); however, this difference was not statistically significant, likely due to the small sample size (Mann–Whitney *U* test, *U* = 101, *Z* = 1.128, *p* = 0.259).

Concerning the examined predictive markers, all tumors were HER2 negative, PD-L1 negative (TPS < 1%), and showed a retained expression of the MMR proteins. CTLA4 showed mostly weak expression in tumor cells in 45% (17/38) of cases. This included 7 cases of well-differentiated, 9 cases of moderately differentiated, and 1 case of poorly differentiated SLCT.

### Molecular findings

**Fig. 2:**
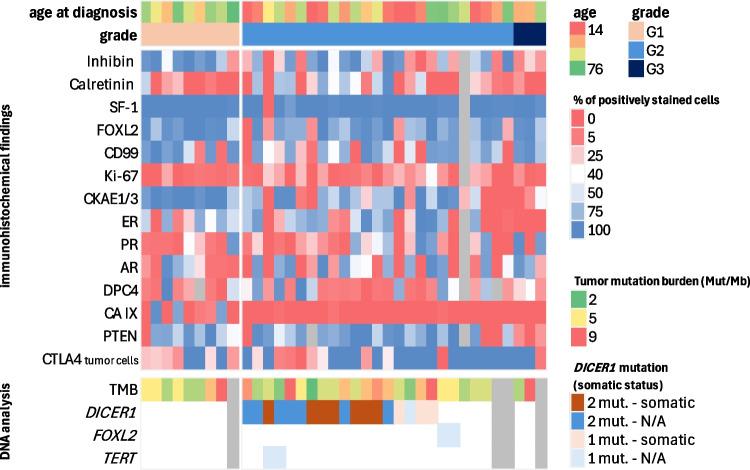
Clinicopathological and molecular findings of Sertoli–Leydig cell tumors. Each column represents a single case. The figure displays only the markers with variable expression (immunohistochemical findings); markers with lack of expression in all cases (such as GATA3, SATB2, napsin A, TTF1, HER2, PD-L1), ARID1A, MMR proteins and MUC4 (only 1 positive case) are not included. Only genes that were mutated in at least two cases are shown

The targeted NGS DNA analysis was successfully performed in 33 SLCTs. The *DICER1* mutation was detected in 54.5% (18/33) of SLCT, all of which were moderately differentiated. Notably, 14 of these tumors harbored two mutations in *DICER1*. The somatic status of *DICER1* was confirmed in 10 *DICER1*^MUT^ tumors, while in the remaining 8 *DICER1*^MUT^ cases it was not possible to confirm or exclude somatic or germline status due to the lack of non-tumor tissue. The *FOXL2* mutation (p.C134W) was detected in 6% (2/33) of SLCT with moderate differentiation, and both of those tumors were *DICER1*^WT^. Both cases showed typical features of moderately differentiated Sertoli–Leydig cell tumors, one with predominantly solid pattern with areas of trabecular formations, and the second with nested type arrangement, both with clusters of Leydig cells. There was also a *TERT* promoter mutation (c.−124C > T) detected in 6% (2/33) of the *DICER1*^MUT^ SLCT. Other pathogenic or likely pathogenic mutations were detected only in individual SLCTs. A detailed list of all the pathogenic or likely pathogenic mutations detected in our cohort is provided in Supplementary Table 3. Moreover, some non-recurrent mutations were also detected in the group of well-differentiated tumors (*n* = 8). An average TMB was 5.8 Mut/Mb (range 2–9; median 6) and no tumor was evaluated as TMB-High (≥ 10 Mut/Mb). The molecular data, including variable protein expression and mutation analysis, are summarized in Fig. 2.

**Fig. 3:**
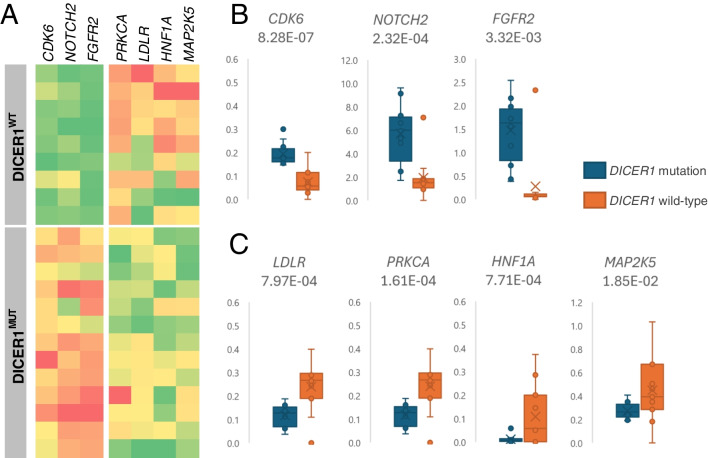
Significantly different mRNA expression between the *DICER1*^MUT^ and *DICER*^WT^ Sertoli–Leydig cell tumors. The transcripts per million (TMP) values of mRNA were normalized to the TPM of the housekeeping gene *HPRT1*. The listed *p*-values were adjusted using the Bonferroni correction. **A** A heat map displaying normalized TPM values in the groups *DICER1*^MUT^ and *DICER1*^WT^ (more intense red indicating higher mRNA levels and green indicating lower levels). **B** Distribution of mRNAs significantly decreased in the *DICER1*^WT^ group (normalized TPM values). **C** Distribution of mRNAs significantly increased in the *DICER1*.^WT^ group (normalized TPM values)

Targeted RNA sequencing was successful in 22 cases. No transcript gene fusion was detected. The expression analysis and comparison between the *DICER1*^MUT^ and *DICER1*^WT^ cases revealed significant differences (Bonferroni correction; adjusted *p*-value < 0.05) in the expression of mRNA in several genes, especially *CDK6*, *PRKCA*, *NOTCH2*, *HNF1A*, *LDLR*, *FGFR2*, and *MAP2K5.* A graphic display of the relevant expression differences is shown in Fig. 3.

## Discussion

SLCTs represent a heterogeneous entity with typical recurrent molecular aberrations occurring only in the subset of moderately and poorly differentiated tumors. Based on this finding, it has been suggested that well-differentiated SLCTs actually represent a different, distinct entity. The *DICER1* mutation seems to be a characteristic, although not specific, feature of SLCT [2, 9]. The reported frequency of this mutation in SLCT is quite variable, with an average prevalence of approximately 65% [25]. In our study, the *DICER1* mutation was present in 54.5% of cases, which is in concordance with other studies. In two previous studies with a higher number of germline *DICER1* carriers, the incidence of *DICER1* mutations was reported to be 88% and 97% [6, 26]. In another study involving 8 pediatric SLCT cases, *DICER1* mutations were found in 100% of the cases, with 5 of these mutations being of germline origin [27]. We did not confirm the germline origin of the *DICER1* mutations in any SLCT; however, in 8 out of the 18 *DICER1*^*MUT*^ SLCT germline status could not be assessed as non-tumor tissue was not available for testing.

The *FOXL2* (p.C134W) mutation is less common in SLCT and is mutually exclusive with *DICER1* mutations. This mutation is regarded as a hallmark alteration of AGCT, but it has also been described in 0–22% of SLCT [1, 2, 6, 7]. We have detected the *FOXL2* mutation in 6% (2 of 33) cases, both were *DICER*^WT^, moderately differentiated, with typical features of Sertoli–Leydig cell tumors and occurred in postmenopausal patients, which is in accordance with literary data. Sometimes there can be difficulty in distinguishing the moderately differentiated SCLT from AGCT, but a thorough investigation and the finding of typical SLCT morphology would help in these cases [2].

Additionally, a *TERT* promoter mutation was discovered in two *DICER*^MUT^ cases in our sample set, both being moderately differentiated. Another molecular alteration, the *TERT* amplification, was found in one of two cases of ovarian SLCT primarily diagnosed as Wilms tumor by Kommoss et al. [28]. While the *TERT* mutation is not typically considered a primary cancer driver event, it has been associated with poorer prognosis in various cancer types, such as AGCT [29]. It has also been described in rare cases of juvenile granulosa cell tumors (JGCT) [30].

Some SLCTs, particularly the well-differentiated tumors, do not show recurrent mutations and are probably driven by another molecular mechanism [2, 9].

*DICER1* mutation disrupts microRNA processing, leading to improper regulation of mRNA and potentially causing abnormal gene expression [2, 31]. Therefore, different expression in *DICER1*^MUT^ and *DICER1*^WT^ SLCT should be expected, which has been confirmed by our results, as well as by Wang et al. [32] in eight SLCTs.

In our cohort of the DICER1^MUT^ cases, we found an increased mRNA expression of oncogenes such as cyclin-dependent kinase 6 (*CDK6*), notch receptor 2 (*NOTCH2)*, and fibroblast growth factor receptor 2 (*FGFR2)*. The aberrant activity of *CDK6*, *NOTCH2*, and *FGFR2* is described and often associated with more aggressive disease phenotypes in certain types of cancer, but it has not yet been studied in SLCT [33–35]. In contrast, an increased expression of protein kinase C alpha (*PRKCA)*, hepatocyte nuclear factor 1 alpha (*HNF1A)*, low-density lipoprotein receptor (*LDLR)*, and mitogen-activated protein kinase 5 (*MAP2K5)* was observed in the *DICER1*^WT^ cases compared to the *DICER1*^MUT^ SLCT. The LDLR primarily plays a role in cholesterol metabolism by mediating the cell uptake of low-density lipoprotein particles, and its overexpression could lead to tumor growth, as has been described in various cancer types such as lung, prostate, and breast cancer [36, 37]. In ovarian epithelial neoplasms, the overexpression of LDLR is associated with cisplatin resistance, and the knockdown of LDLR reduces tumor growth by suppressing autophagy associated with the PI3K/AKT/mTOR pathway [38]. The relationship between *LDLR* and SLCT has not yet been described, and our results suggest that there could be increased cell uptake of low-density lipoproteins in *DICER1*^WT^ SLCT. The function and relationships between ovarian cancer and other upregulated mRNA genes (*PRKCA*, *HNF1A*, and *MAP2K5*) are not clear and further investigation is necessary. Wang et al. [32] analyzed the global miRNA and gene expression in SLCTs with and without *DICER1* hotspot mutations and demonstrated that *DICER1* hotspot mutations were associated with the global reduction of 5p-derived miRNAs in ovarian SLCT. They also compared mRNA expression (whole transcriptome) between four *DICER1*^MUT^ and four *DICER1*^WT^ SLCTs and detected 1,396 differentially expressed genes in these two cohorts, with enrichment of genes regulating cell survival, proliferation, and development. Their analysis was performed on a small cohort of cases, with further mRNA investigation based on these initial findings. They used a different approach to RNA expression analysis compared to ours, specifically the Illumina Whole-Genome DASL Microarray Analysis which is more complex but could be less sensitive than targeted RNA sequencing. These factors may therefore account for the differences, with Wang et al. [32] identifying *FGFR2* as differentially expressed, but without detecting any of the other genes which we observed to vary between *DICER1*^MUT^ and *DICER1*^WT^ SLCTs.

Most SLCTs are diagnosed in the early stages of the disease, so surgical treatment represents the main therapeutic approach [39]. Advanced stages or relapsing cases can be treated by adjuvant chemotherapy, but knowledge of new therapeutic options for these tumors is limited [40]. We performed targeted RNA sequencing mainly to identify targetable fusions of the pan-markers in solid cancers such as *NTRK1*/2/3, *ALK*, *ROS1*, and *RET*. No targetable fusion was detected in our cohort of SLCT.

We also explored the expression of selected possible predictive markers, including PD-L1, CTLA4, HER2, and MMR proteins. The significance of immune checkpoint inhibitors in cancer immunotherapy has been steadily increasing and is becoming more and more important [41, 42]. So far, no study has explored PD-L1 or CTLA4 expression in ovarian SLCT. We did not find any PD-L1 positivity in the tumor cells. CTLA4 expression was seen in 43% of cases, mostly of weak to medium intensity, and without association with grade of differentiation. However, CTLA4 is not currently a clinically established predictive marker for immunotherapy. Several studies found CTLA4 expression in a variety of tumors such as breast, lung, and cervical cancer, and in hematological malignancies and ovarian, uterine, and cervical cancer cell lines [43–48]. Concerning the expression of CTLA4 in other ovarian sex cord-stromal tumors, we found positivity in 69% of AGCT in our previous study [49]. Overall, the results of studies investigating the prognostic significance of CTLA4 are unsatisfactory, since some of them show a positive relationship, others negative, and the rest did not find any association between the expression and prognosis [44, 48, 50, 51].

The HER2 expression in ovarian sex cord-stromal tumors was examined only in granulosa cell tumors (GCT), but not in SLCT [49, 52–57]. All but two studies focusing on HER2 in GCT (including our previous study) showed no HER2-positive cases. Only two studies found several cases that were immunohistochemically HER2 positive, but a subsequent examination performed in one of these cases did not reveal *HER2* amplification [56, 57]. We did not find any HER2 positivity in SLCT.

MMR deficiency and/or high microsatellite instability are used to predict the response to immune checkpoint inhibitor therapy in solid tumors [58]. So far, only two studies have explored the expression of MMR proteins in sex cord-stromal tumors, specifically in AGCT, and no case showed MMR protein expression deficiency [59]. In our study, all cases were MMR proficient. However, the molecular results revealed one case with a pathogenic *MSH6* mutation along with two common cancer-driver mutations in *DICER1.*

Another marker that was examined for the first time in our study was PTEN expression. We found a loss of PTEN expression in 14% (5/35) of SLCT, but no case showed molecular alterations in the coding sequence of the *PTEN* gene. The function of PTEN can be affected by a wide range of genetic and epigenetic changes or modulated by post-transcriptional or post-translational regulations—these mechanisms are therefore probably involved in the observed loss of PTEN expression. Moreover, there are currently no uniform scoring criteria for PTEN expression, or a validated test which could predict the lack of PTEN function [60]. There are some studies in which the loss of expression/presence of *PTEN* mutation is regarded as a predictive biomarker, but the precise role of PTEN as a potential prognostic and/or predictive biomarker has yet to be elucidated [60, 61].

The differential diagnosis of SLCT can be difficult in some cases and includes mainly the other types of sex cord-stromal tumors, such as JGCT and AGCT, but also tumors of other histogenesis, such as the hypercalcemic type of small cell carcinoma, primary or metastatic endometrial stromal sarcoma, undifferentiated carcinoma, and endometrioid carcinoma. SLCT generally expresses the “traditional” sex cord markers such as calretinin, inhibin A, CD99, FOXL2, and SF1. Inhibin and calretinin are positive in a majority of SLCT, with a typically stronger expression in Leydig cells compared to Sertoli cells (inhibin 64–100%, and calretinin 48–100%) [7, 10–14]. SF1 expression was mostly described in Sertoli cells and Sertoli cell tumors, where it is present in all reported cases [14, 18]. The expression of CD99 and FOXL2 in SLCT ranges from 59–100% and 50–100%, respectively [14, 16–19]. This data is in accordance with our results, which showed the expression of inhibin, calretinin, CD99, FOXL2, and SF1 in 94%, 58%, 81%, 97%, and 97% of cases. All cases showed the expression of at least three of these markers. In general, the sensitivity of these markers seems to be high, but their potential use in differential diagnosis with other sex cord-stromal tumors is limited, and molecular testing should be used in diagnostically problematic cases.

A variety of sex cord-stromal tumors, including SLCT, have also been reported to express cytokeratins, such as CKAE1/3. Goulvin et al. [7] found keratin positivity in 82% (14/17) cases. Our results showed mostly dot-like cytoplasmic positivity of CKAE1/3 in 83% of cases. Although SLCTs show a rather higher CKAE1/3 expression than AGCT (where it has been described in 26–58% of cases), in common practice this finding is not very useful for differential diagnosis [11, 49, 62–64].

Hormonal receptors can be of both diagnostic and therapeutic significance. AR receptor expression has not yet been investigated on a larger sample set, and only one study investigated ER and PR expression in ovarian SLCT. They found ER expression in 79% and PR in 86% of cases [65]. Our results showed expression of ER in 72%, PR in 58%, and AR in 67% of cases.

The expression of Ki67, p53, and p16 has so far been described in SLCT only in rare case reports [15, 66, 67]. The median proliferation index (Ki67) observed in our study was 3 (range 0–38), with a mean value of 8 (SD 9.4). There were differences between the well-differentiated and moderately/poorly differentiated SLCTs, but this finding did not reach statistical significance due to the insufficient number of cases. The p53 expression was investigated only in one study including four sex cord-stromal tumors: two SLCT (one well-differentiated and one poorly differentiated) and two AGCT. The authors found some p53 positivity in the Sertoli cells component which; however, did not reach the criteria for overexpression [15]. Our results did not reveal any case with an aberrant expression of p53, or diffuse block type 16 positivity.

The expression of CAIX, GATA3, SATB2, napsin A, MUC4, TTF1, DPC4, and ARID1A has not been investigated in SLCT to date. The expression of CAIX, so far mostly studied in malignant epithelial tumors, has been associated with a worse prognosis in several carcinomas, including breast cancer, gastric cancer, and some others [68, 69]. In our study, CAIX expression was found in 14% of SLCTs, all of which were well-differentiated tumors, that have excellent prognoses This finding does support the thesis that well-differentiated SLCTs are a distinct tumor type compared to moderately and poorly differentiated tumors. Surprisingly, one moderately differentiated SLCT showed weak to moderate MUC4 expression. We did not find a simultaneous *MUC4* mutation in this case, but literary data describes an *MUC4* mutation in one case of a moderately/poorly differentiated tumor in a study of 19 SLCT [70]. Concerning the other markers examined, none of our cases showed positive staining with SATB2, napsin A, GATA3, or TTF1 antibodies, which can be useful with respect to differential diagnosis.

The differential diagnosis of SLCT can be difficult in some cases, especially when the moderate/poorly differentiated cases are considered. Especially tumors of different histogenesis have to be ruled out, such as epithelial tumors, where the positivity of EMA and negativity of sex cord-stromal markers would be helpful. Depending on the age of the patient, germ cell tumors can also come into differential diagnosis, especially yolk sac tumors, in which case SALL4 negativity favors SLCT, but one has to remember that focal AFP expression is common in SLCT [20]. Differentiation from other sex cord-stromal tumors can sometimes also be challenging, and using molecular testing can be of use in these cases: finding *DICER1* mutation would support the diagnosis of SLCT, while the presence of *AKT1* or *GNAS* mutations would support a diagnosis of juvenile granulosa cell tumor [71]. The finding of *FOXL2* mutation combined with typical morphological features would support AGCT since a small amount of SLCT also harbors this mutation. Finding other mutations in genes such as *CTNNB1* or *APC* would favor microcystic stromal tumors, and the presence of *GLI2* fusion suggests sclerosing stromal tumors [71]. The role of molecular testing has become more and more important when diagnosing these tumors. However, it should be emphasized that morphology is key in diagnosing these tumors, and a thorough examination is always necessary.

We acknowledge that there are some limitations of our study, the main one being related to the use of tissue microarrays (TMAs). Although widely utilized, particularly in studies involving larger cohorts, this approach theoretically raises the risk of either underestimating or overestimating the immunohistochemical scoring. The mRNA expression analysis also had several limitations: a targeted panel of selected genes was used, the sample size of our cohort was limited, and post-transcriptional or post-translational regulations were not considered. This indicates a need for further, more comprehensive investigation into mRNA expression in SLCT.

## Conclusion

Our study provides a comprehensive characterization of the molecular landscape and immunohistochemical features of SLCT. We confirmed that *DICER1* and *FOXL2* mutations are mutually exclusive and are restricted to moderately and poorly differentiated tumors. We also describe a *TERT* promoter mutation in these tumors. Additionally, we identified significant differences in mRNA expression between the *DICER1*^MUT^ and *DICER1*^WT^ SLCT. Taken together, our results support the view that well-differentiated tumors are different from moderately and poorly differentiated ones, and probably represent a different entity on the molecular level. Concerning the possible predictive markers, our results show that SLCTs are microsatellite stable, do not express PD-L1, and are HER2 negative.

## Supplementary Information

Below is the link to the electronic supplementary material.

Supplementary file1 List of antibodies (DOCX 20 KB)

Supplementary file2 List of genes included in the DNA and RNA NGS targeted panels (XLSX 20 KB)

Supplementary file3 All detailed pathogenic or likely pathogenic mutations detected in the cohort of Sertoli–Leydig cell tumor (XLSX 17 KB)

## Data Availability

All data generated or analyzed during this study is included in this published article and its Supplementary information files.
