# Supplementary material for: A molecular and immunohistochemical study of 37 cases of ovarian Sertoli–Leydig cell tumor
Source: Virchows Arch. 2024 Nov 27;487(1):127–40. doi: 10.1007/s00428-024-03984-5 (PMC12289768; doi:10.1007/s00428-024-03984-5)
Supplement: Supplementary file 1 — Supplementary file1 List of antibodies (DOCX 20 KB) [file 428_2024_3984_MOESM1_ESM.docx]

Supplementary table 1. Overview of the used immunostains and their main characteristics.

| **Marker** | **Supplier** | **Clone** | **Primary dilution** | **Antigen retrieval** | **Detection** | **Evaluated expression** |
| --- | --- | --- | --- | --- | --- | --- |
| **FOXL2** | Novus Bio | Polyclonal Ra | 1:200 | - | Ventan Optiview Amplification Kit, Ventana | Nuclear |
| **SF1** | Abaam | EPR 19744 | 1:400 | HIER (pH 9) | EnVision FLEX, Dako + Linker | Nuclear |
| **CD99** | DCS | EPR 3097Y | 1:200 | - | Ventan Optiview Amplification Kit, Ventana a | Cytoplasmic |
| **Inhibin A** | Dako | R1 | RTU | HIER (pH 9) | EnVision FLEX, Dako | Cytoplasmic |
| **Calretinin** | Dako | DAKCalret | 1:00 | HIER (pH 9) | EnVision FLEX, Dako + Linker | Cytoplasmic |
| **ER** | Zytomed | SP1 | 1:200 | - | Optiview Amplification Kit, Ventana | Nuclear |
| **PR** | Novocastra | 16 | 1:100 | - | Optiview Amplification Kit, Ventana | Nuclear |
| **AR** | Neo Markers | AR 441 | 1:25 | - | Ventan Optiview Amplification Kit, Ventana | Nuclear |
| **CKAE1-3** | Dako | AE1/AE3 | 1:200 | HIER (pH 9) | EnVision FLEX, Dako | Membranous and cytoplasmic |
| **Ki67** | Dako | MIB-1 | 1:100 | - | UltraView Detection Kit, Ventana | Nuclear |
| **P53** | Dako | DO-7 | 1:400 | - | UltraView Detection Kit, Ventana | Cytoplasmic and nuclear |
| **P16** | DB Biotech | R15-A | 1:800 | - | Optiview Amplification Kit, Ventana | Cytoplasmic and nuclear |
| **GATA3** | Cell Marque | L50-830 | 1:400 | HIER (pH 9) | EnVision FLEX, Dako + Linker | Nuclear |
| **ARID1A** | Abcam | EPR 13501 | 1:1000 | HIER (pH 9) | EnVision FLEX, Dako | Nuclear |
| **Napsin A** | Novocastra | IP64 | 1:200 | HIER (pH 9) | EnVision FLEX, Dako + Linker | Cytoplasmic |
| **SATB2** | Cell Marque | EP281 | 1:400 | HIER (pH 9) | EnVision FLEX, Dako | Nuclear |
| **MUC4** | Bio SB | EP256 | 1:400 | HIER (pH 6) | EnVision FLEX, Dako | Cytoplasmic |
| **TTF-1** | BioCare | SPT24 | 1:200 | - | Optiview Amplification Kit, Ventana | Nuclear |
| **DPC4** | Zeta Corporation | B-8 | 1:200 | HIER (pH 9) | EnVision FLEX, Dako | Nuclear |
| **CAIX** | Novus Bio | Polyclonal Ra | 1:1600 | HIER (pH 9) | EnVision FLEX, Dako | Membranous |
| **CTLA4** | Bio SB | BSB - 88 | 1:25 | - | Optiview Amplification Kit, Ventana | Membranous and cytoplasmic |
| **PTEN** | Dako | 6H2.1 | 1:200 | HIER (pH 9) | EnVision FLEX, Dako | Cytoplasmic and nuclear |
| **HER2** | Roche | - | RTU | - | UltraView Amplification Kit, Ventana | Membranous |
| **PD-L1** | Dako | 22C3 | 1:40 | - | Optiview Amplification Kit, Ventana | Membranous and cytoplasmic |
| **MLH1** | Dako | ES 05 | RTU | HIER (pH 9) | EnVision FLEX, Dako + Linker | Nuclear |
| **PMS2** | Dako | EP 51 | RTU | HIER (pH 9) | EnVision FLEX, Dako + Linker | Nuclear |
| **MSH2** | Dako | FE 11 | RTU | HIER (pH 9) | EnVision FLEX, Dako + Linker | Nuclear |
| **MSH6** | Bio SB | EP49 | 1:400 | HIER (pH 9) | EnVision FLEX, Dako + Linker | Nuclear |
